# Marker Substances in the Aroma of Truffles

**DOI:** 10.3390/molecules27165169

**Published:** 2022-08-13

**Authors:** Ruben Epping, Lilly Bliesener, Tilman Weiss, Matthias Koch

**Affiliations:** 1 Division of Organic Trace Analysis and Food Analysis, Bundesanstalt für Materialforschung und -Prüfung, 12489 Berlin, Germany; 2 Sglux SolGel Technologies GmbH, 12489 Berlin, Germany

**Keywords:** truffle, volatile organic compounds, gas chromatography, mass spectrometry, canine olfactometry

## Abstract

The aim of this study was to identify specific truffle marker substances within the truffle aroma. The aroma profile of different truffle species was analyzed using static headspace sampling with gas chromatography mass spectrometry analysis (SHS/GC-MS). Possible marker substances were identified, taking the additional literature into account. The selected marker substances were tested in an experiment with 19 truffle dogs. The hypothesis “If trained truffle dogs recognize the substances as supposed truffles in the context of an experiment, they can be regarded as specific” was made. As it would be nearly impossible to investigate every other possible emitter of the same compounds to determine their specificity, this hypothesis was a reasonable approximation. We were interested in the question of what it is the dogs actually search for on a chemical level and whether we can link their ability to find truffles to one or more specific marker substances. The results of the dog experiment are not as unambiguous as could have been expected based on the SHS/GC-MS measurements. Presumably, the truffle aroma is mainly characterized and perceived by dogs by dimethyl sulfide and dimethyl disulfide. However, as dogs are living beings and not analytical instruments, it seems unavoidable that one must live with some degree of uncertainty regarding these results.

## 1. Introduction

Truffles were already known as a delicacy among the Romans, Greeks, and Babylonians in ancient times [1]. They were considered a mysterious food because their origin was unknown [2]. A student of Aristotle’s assumed that truffles were formed from autumn rain or lightning strikes [3]. Today, we know that truffles are hypogaric fungi belonging to the Tuber genus [1,4]. They live in symbiosis with plants by forming a widely branched network of hyphae connected to the root cortex of the plants [1,2,3,5]. Due to the large-scale, structural nature of the truffle, there is the possibility of a beneficial, mutual exchange of resources, such as water, nutrients, or photosynthesis products, between the truffle and the host tree [3,6]. During the ripening process, the so-called asocarp (fruiting body) is formed from the hyphae of the truffle [1]. It is known that the formation of the fruiting body would not be possible without the symbiosis and that at least some truffle species have an active life cycle and metabolism [1,3].

The number of truffle species existing worldwide is between 180 and 240 [3]. About 30 different types of truffles are commercially marketed. Some are particularly popular and are sought after or cultivated and harvested in numerous countries [7].

The most expensive and sought-after truffle is *Tuber magnatum pico*. It is often referred to as the white Alba or Piedmont truffle. *Tuber melanosporum*, the black truffle, is the most cultivated truffle. It is also known as the Périgord truffle because of its original origin, and it is almost as popular as the white truffle [8,9]. The summer truffle, *Tuber aestivum*, is also referred to as the “poor relative” among truffles as it is widespread but has a much lower intensity of taste and smell [10]. The summer truffle is harvested continuously from May through to the winter months. It is characterized by its black exterior and its light brown flesh with white veins.

In addition to these three most-traded truffles, there are a few other truffles that are also used for culinary purposes. Some of them are also used for the purpose of counterfeiting higher quality truffles. The Asia truffle, *Tuber indicum*, is visually similar to *Tuber melanosporum*. The fruiting body is dark brown in color and has an almost tasteless, black-brown flesh with small white veins [3]. Tuber indicum is nevertheless exported to Europe and often used to counterfeit *Tuber melanosporum*. For this, the Asia truffle is coated with artificial truffle oil to imitate the aroma [3].

Today, around 80% of the truffles used are grown by inoculating young trees with truffle spores [1]. For a long time, truffle cultivation was conducted by planting the acorns or tree seedlings of truffle-producing oak trees in open spaces. Nowadays, trees or shrubs inoculated with truffle spores are almost exclusively planted [8]. The particularly valuable *Tuber magnatum* however cannot be bred and cultivated as productively as many other truffle varieties and is therefore still mainly harvested in the wild form.

One reason for the high price of truffles, in addition to their rarity and lengthy cultivation, is the difficult harvesting conditions. To harvest truffles, trained truffle dogs or, less frequently, pigs are used. They can smell the volatile aromatic substances contained in the truffle over long distances and thus localize them in the ground [3]. The dogs do not naturally eat truffles and must therefore first be trained on the smell [11]. The sense of smell is the most sensitive and important of all senses in dogs [12]. The lowest proven concentration limit in dogs is in the ppt range [13]. Many analytical devices, for comparison, have their concentration limits in the ppm to ppb range.

Training to become a truffle dog must take place very soon after the animal is born and lasts at least a year. At the beginning of the training, it is important that the dog perceives the truffle as a toy and develops joy in handling it. After habituation to the truffle, further requirements are added. An example is truffle catching. For lasting success and learning progress, it is imperative that the exercises are repeated daily. If these function without problems, the sense of smell is trained in the following training section. For example, handlers train with the dogs in tall grass. The dog is thus forced to use its sense of smell instead of its eyes to find the truffle. As soon as the dog can find the truffle, it can start searching in the ground. This step is the most crucial within the training series as the dog should not follow the smell of the owner, but that of the truffle. After the end of the training, it is important to search for truffles with the dog in the forest or on a farm in order to apply what it has learned. However, it is essential to ensure that the dog takes breaks from time to time and is not stressed or tired. Otherwise, he loses the joy of searching [11].

According to the current state of research, the smell of truffles is mainly caused by bacteria and microbes specific to truffles, such as yeasts and filamentous fungi. They originate from the soil and produce odorous volatile organic compounds (VOCs) [3,4,5]. Their exact composition varies within the truffle species and the possible influencing factors are not exactly known. The smell of the truffles naturally serves to reproduce or spread. The intensive aroma attracts animals that eat the fruiting body and thus spread the truffle spores that are attached to them [3,14].

Due to their fascinating nature, truffles have been the subject of research for many years. In particular, the aroma of truffles has been examined in numerous studies. Both the truffle itself and the truffle products were analyzed [15,16]. The methods used range from various gas chromatographic techniques to electronic noses [17,18,19,20,21]. In particular, the combination of gas chromatography and mass spectrometry (GC-MS) with a headspace sample application system was used in a wide variety of combinations [22,23,24]. The focus was primarily on volatile organic sulfur compounds (VSCs). This class of substances can be perceived by dogs and humans even in very low concentrations [13]. Different representatives of this class of substances have been found in different truffles [22]. The composition and concentration also vary with the origin and the cultivation conditions of the truffle [4]. Dimethyl sulfide (DMS) and 2,4-ditihapentane (*in Tuber magnatum pico*) have been especially widely reported, and it is suggested that dogs can use these compounds to detect truffles [25,26,27].

In particular, a study by Talou et al., testing various volatile organic compounds using truffle dogs [28], has excited our interest. Talou et al. found that dogs detect samples of DMS as truffles. In our opinion, however, this study has some weaknesses. The described used concentration of the analytes of 300 mL/L dissolved in sunflower oil appears to be exorbitantly high. In addition, no precise test procedure of the experiments is described, and the number of the tests or the participating dogs is not specified. A statistical test evaluation did not take place either. DMS was also the only sulfur-containing compound tested; so, it cannot be ruled out that other VSCs would also have led to a positive result. For these reasons, we decided to conduct further research in this direction.

Based on this state of the research, the aim of this work was the characterization of the truffle aroma and the identification of the marker substances which are characteristic for truffles in general. For this purpose, the aroma profiles of different types of truffles were analyzed by static headspace (SHS) sampling with GC-MS analysis. Possible marker substances were then identified from the measurement results and by taking the additional literature into account. To check whether the selected substances are actually characteristic of truffles, the following hypothesis was put forward: “If trained truffle dogs recognize the substances as supposed truffles in the context of an experiment, they can be regarded as specific”. This hypothesis was tested in an experiment with truffle dogs from 19 different truffle farms. It is known that truffle dogs, trained to find a specific variety of truffle, can also find other varieties of truffles. We were interested in the question of what it is the dogs actually search for on a chemical level and whether we can link their ability to find truffles to one or more specific truffle marker substances.

## 2. Results

### 2.1. Key Aroma Components Known from Literature

It is known that the aroma of truffles is largely determined by the VOCs they contain. The majority of these compounds can be assigned to the substance class of alcohols, aldehydes, ketones, aromatics, and sulfur compounds [3]. The odors of the individual components vary greatly between, for example, earthy, vanilla-like, garlic-like, cheesy, pungent, dusty, or petrol-like. The special aroma and the unique taste of the truffle varieties are determined by the combination of the volatile compounds [3].

In the literature, when describing the aroma profile of truffles, a distinction is made between the marker substances that are characteristic of a certain truffle variety and the marker substances that influence the aroma but are contained in almost all truffle species. However, this does not mean that the specific marker substances do not also occur in other truffle varieties. What is meant is that the specific marker substances in the respective truffle variety occur in high concentrations or contribute a large part to the aroma profile of this variety.

In the course of this work, the four different types of truffles, *Tuber magnatum pico*, *Tuber melanosporum*, *Tuber aestivum*, and *Tuber indicum*, were examined. An overview of the aroma components mentioned in the literature and specific to these varieties is given in Table 1.

### 2.2. GC-MS Measurement

The measurements using the SHS method are particularly interesting for this study as an aliquot of the aroma is injected directly into the GC-MS. Of all possible injection methods, this corresponds most closely to the aroma composition that a truffle dog or human would smell. Optimizing the method showed that the higher the equilibration temperature, the more numerous the peaks with higher intensity the chromatogram contained. However, to come as close as possible to the realistic application, the equilibration temperature was left at a value of 40 °C.

**Figure 1:**
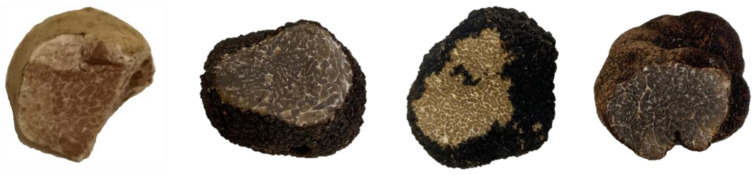
Investigated truffle species, from left to right: *Tuber magnatum pico, Tuber melanosporum, Tuber aestivum*, and *Tuber indicum*.

**Figure 2:**
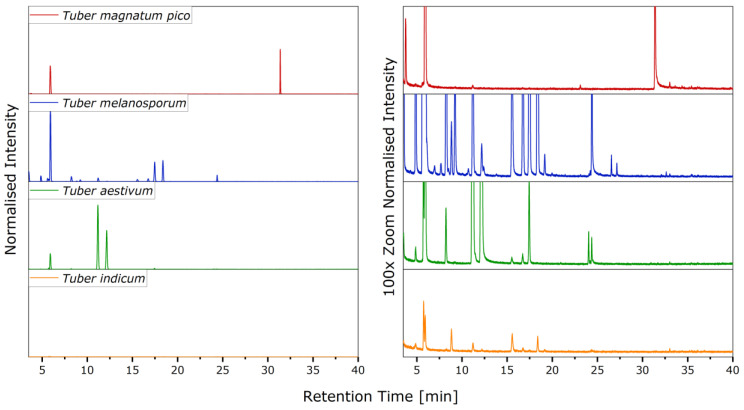
SHS/GC-MS total ion chromatograms of the examined truffles.

The truffle varieties shown in Figure 1 were analyzed using SHS/GC-MS measurements, obtaining the chromatograms in Figure 2.

In general, Figure 2 shows that all the investigated types of truffles have different aroma profiles. The significantly larger number of signals in the *Tuber melanosporum* sample is particularly striking. This indicates a particularly high variety of substances in this variety. The intensities of the signals differ not only within a chromatogram, but also between the different types. The highest intensities occur with *Tuber melanosporum* and *Tuber aestivum*, the lowest with *Tuber indicum*. From this, it can presumably be deduced that the intensity of the aroma is lowest in *Tuber indicum*.

A calibration was not carried out as it is the relative composition that is of importance for this study. An absolute quantification would be of little value in this investigation context, because it would only reflect the concentration of the compounds in the gas phase of a headspace sample vial under controlled conditions. No conclusions could be drawn from this as to the concentrations found in a natural environment. The purpose of this analysis was to identify and roughly estimate the importance of VOCs in the truffle aroma. An exact quantification would not be useful for this as the aroma concentrations can be expected to vary within different samples of the same truffle variety. In addition, some of the compounds are not commercially available as pure substances. Nevertheless, the measurements can be compared with each other because the same measurement method and the same weights were used. In addition, the relative differentiation of individual compounds in a sample is possible semi-quantitatively as the sensitivity of the detector for individual compounds is between about 0.8 and 1.2 [29].

**Figure 3:**
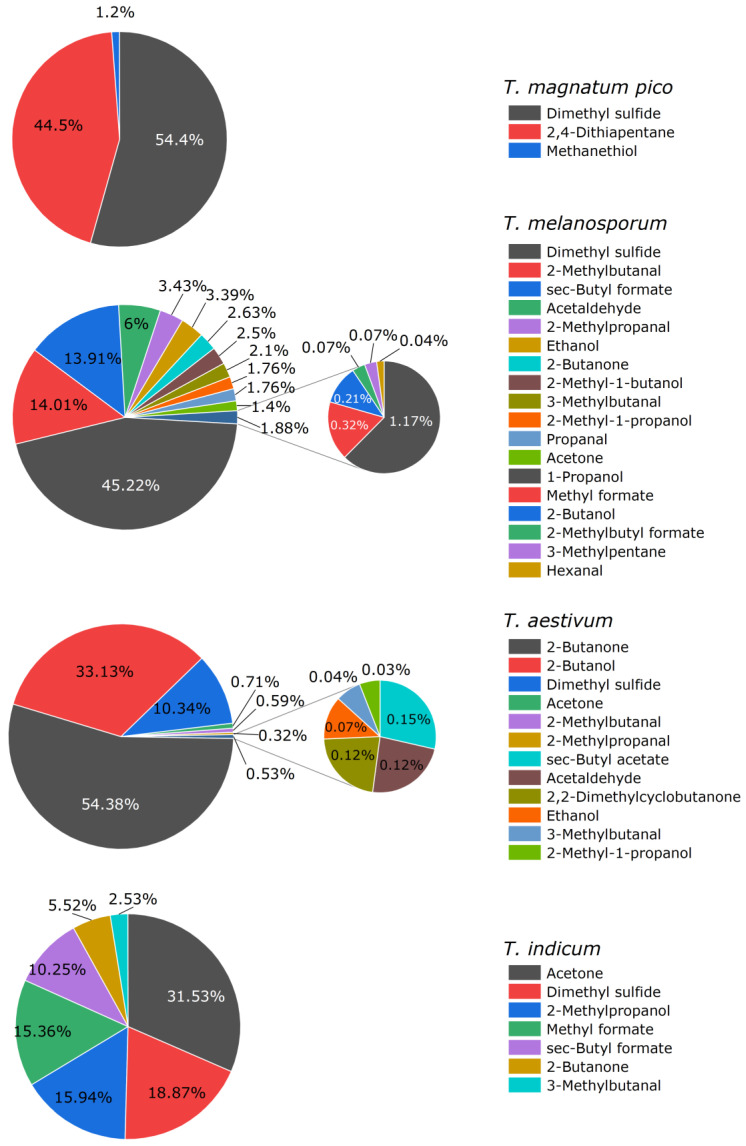
Aroma compounds of truffle samples as peak area percentage of the total ion chromatogram of SHS/GC-MS analyses.

A total of 26 different aroma substances could be identified. An overview of all the substances can be seen in Table A1 in the Appendix A and in Figure 3. The pie charts show the composition of each type of truffle as their percentage of the total chromatogram.

A particularly important aroma component of truffles, as has already been described in the literature, seems to be dimethyl sulfide (DMS). It is the only substance contained in all four types of truffles. However, the respective percentage of the entire chromatogram varies greatly. In the samples of *Tuber magnatum pico* and *Tuber melanosporum*, it is present at 54% and 45%, respectively, *in Tuber aestivum* and *Tuber indicum* only at 10% and 19%, respectively. From this, it can be concluded that DMS is one of the main aroma components, especially in *Tuber melanosporum* and *Tuber magnatum pico*.

In the relevant literature, dimethyl disulfide (DMDS) and 1-octen-3-ol have also been mentioned as VOCs that are present in all or many truffle varieties. Neither of the two substances could be identified in this series of measurements.

In the sample from *Tuber magnatum pico*, 2,4-dithiapentane, DMS, and a small amount of methanethiol could be identified. 2,4-Dithiapentane is one of the substances described in the literature as specific marker substances. As it was contained in the sample at a percentage of 44%, it can be assumed that, alongside DMS, it is a main aroma component of this truffle variety. The other two substances described in the literature as specific marker substances (Table 1) could not be detected in this measurement.

In the *Tuber melanosporum* sample, a total of 18 different compounds could be identified. The diagram shows that the *Tuber melanosporum* sample contains three other prominent substances in addition to the main aroma component DMS. These substances are 2-methylbutanal (14%), sec-butyl formate (14%), and acetaldehyde (14%). The other 14 aroma components make up only a minor amount of the chromatogram. Both 2-methylbutanal and methyl formate have been mentioned in the literature as specific flavoring substances of *Tuber melanosporum*. 3-Methylbutanal, which was also described as being specific for *Tuber melanosporum*, was identified in this sample at a level of 2.1%. Anisole, which is listed in the literature as another specific marker substance, was not identifiable in this measurement. All three marker substances specific for *Tuber melanosporum* could also be partially detected in the samples from *Tuber aestivum* and *Tuber indicum*. In the *Tuber aestivum* sample, however, the percentages of these substances were very low, at 0.04% for 3-methylbutanal and 0.59% for 2-methylbutanal. In the sample from *Tuber indicum*, on the other hand, sec-butyl formate was present with a very large proportion at 10%, and 3-methylbutanal was present with an almost identical proportion to that in *Tuber melanosporum* at 2.5%. Based on these measurement results, it is questionable whether sec-butyl formate and 3-methylbutanal can actually be designated as specific marker substances for *Tuber melanosporum*, as described in the literature. Perhaps, like DMS and DMDS, these should be counted among the substances that influence the aroma profile in several types of truffles.

As described in the literature, the aroma of *Tuber aestivum* is presumably dominated by 2-butanone and 2-butanol. DMS has a percentage of 10% and all the other nine substances identified have a percentage between 0.03% and 0.7% of the total chromatogram. Although 2-butanone and 2-butanol were also detected in the samples from *Tuber melanosporum* and *Tuber indicum*, the percentages were much lower.

A total of seven different substances were identified in the *Tuber indicum* sample. 3-Methylanisole, which is referred to in the literature as a specific marker substance, was not one of them. What is striking when looking at the diagram is that acetone makes up the largest part of the chromatogram at 33%. All the other components, such as methyl formate and 2-methylpropanol, could also be detected in *Tuber melanosporum*. Although the percentages differ and the overall aroma intensity is much lower, there is a clear similarity between the aroma components that occur.

In addition to the four types of truffles, a truffle oil with black truffle was examined. Small pieces of truffle were visible on the bottom of both truffle oils. However, the term “flavours” was also included in the list of ingredients. It can therefore be assumed that the oil consists of a mixture of synthetically added and natural flavorings. The constituents of the aroma were methanethiol (33.2%), 2,4-dithiapentane (30.9%), *p*-dithiane-2,5-diol (14.4%), ethanol (12.3%), 1,3-octadiene (4.6%), DMS (2.9%), nonanal (1.1%), 2-butanone (0.4%), and 3-methylbutanal (0.2%).

The measurement results show that the truffle oil contains both identical and different aroma components in comparison to the fresh truffle sample. However, the occurring intensities and the number of components is similar. Like the truffle samples, DMS could also be identified as an aroma component in the oil, although the amount was significantly lower. It is particularly striking that this truffle oil is named “black truffle oil” and that supposed truffle pieces could also be seen in the bottle, but it does contain 2,4-dithiapentane. This substance could be detected with a percentage of 31% and is a characteristic marker substance for *Tuber magnatum pico*. In addition, methanethiol was identified with a percentage of 33%. This substance was also only detected in the sample from *Tuber magnatum pico*. From an analytical point of view, it is more the case that this truffle oil imitates white truffle. All other substances, except for DMS and ethanol, were not identified in any truffle sample. The *p*-dithiane-2,5-diol stands out in particular. It accounts for 15% of the total chromatogram; so, the industry probably counts it as an important truffle flavor mimicking component that does not naturally occur in truffles.

In summary, it can be said that DMS is the only aroma component that could be detected in all the samples. This suggests that DMS could be a marker substance for truffles in general. Due to the different amounts of DMS, it can be concluded that the influence of this substance on the aroma profile is not identical in all truffle varieties.

With this approach, it should be noted that the proportions of the peak areas of the identified substances cannot necessarily be compared with each in terms of aroma composition. One reason for this is the different odor thresholds of all the substances. The substances with a low concentration can also make a major contribution to the perception of the aroma.

### 2.3. Selection of Possible Marker Substances

To determine which substances are specific for truffles, the hypothesis was constructed that a substance can be classified as specific if it is recognized as a putative truffle by trained truffle dogs. This hypothesis was put forward for practicable reasons. In order to determine the specificity of a VOC for truffles, not all the other possible emitters of the same VOC, such as other fungi, roots, trees, animals, fruits, or anthropogenic sources, can be checked for it.

As not all the identified substances could be tested in the experiment with the truffle dogs, the selection of possible marker substances shown in Table 2 was made based on the literature and the measurement results obtained.

A previous study [28] was also considered in which the following substances were tested as part of a similar experiment with dogs: acetaldehyde, DMS, 2-methylpropanal, acetone, ethanol, 2-butanone, 2-methylbutanal, and 2-methylpropanol. The study authors identified DMS as the only compound recognized by the animals [28]. However, this study has some weaknesses in our opinion, as already described in the Section 1 above. To enable a correlation of the results with this previous study, in addition to DMS, two of the compounds excluded by this study as marker substances (2-butanone and 2-methylbutanal) were selected again within the scope of our test series.

The order of the substances was based on the perceived odor intensities and based on the odor thresholds for humans given in the literature. In addition, care was taken to ensure that not all the sulfur compounds were tested one after the other.

As the substances are volatile compounds, they were dissolved in odorless and tasteless medicinal white oil. The pure white oil was also used as a negative control.

The positive control is listed in the second position. The truffle oil was selected because, according to the measurement results, it contained aroma components from both *Tuber magnatum pico* and *Tuber melanosporum*. These two types of truffles are the most popular in the world and should therefore be recognized by many truffle dogs. In addition, it is described in the secondary literature that, among other things, this truffle oil (as well as truffle oils of this type in general) is used to train truffle dogs [32,33].

Phenylacetaldehyde was specified as the third component. According to literature, this is mainly contained in *Tuber melanosporum* and *Tuber aestivum*. Although it could not be identified within the scope of the measurements carried out, acetaldehyde was used as a similar compound in the study already mentioned [28] and was thus also identified as a potential marker substance.

Fourth is 1-octen-3-ol. This compound is referred to in the literature as an important aroma component of many truffle varieties and was identified in the measurements of the truffle oil. It is described in the literature that 1-octen-3-ol is only formed in larger concentrations during ripening or overripening after the truffle has been harvested and thus may indicate truffle ripeness.

3-Methylthiophene in the fifth position was chosen because, according to the literature, it is a marker substance specific to *Tuber borchii* (spring truffle). The substance could not be detected in the measurements carried out because this type of truffle was not part of the series of measurements. Nevertheless, this substance was included to test as broad a spectrum of different marker substances as possible.

2-Butanone in sixth place was chosen because this substance was detectable in all samples but *Tuber magnatum pico*.

Nonanal is listed in the seventh position because it is referred to in the literature as an aroma component of truffles in general. Although this substance could not be identified by us in any of the truffle samples, it could be identified in the truffle oil. This indicates that the substance is also one of the marker substances important for the truffle aroma in the industry.

DMDS in eighth place was not detected; however, the literature shows it often co-occurs with DMS in very small amounts with a similar odor. It may actually be present in the samples but was not detected with the SHS method due to its low concentration. To test whether truffle dogs may be attracted to this substance in addition to DMS, it was included in the study.

2- and 3-Methylbutanal, in the ninth and eleventh positions, were chosen because both substances are often mentioned in the literature as the main aroma component of *Tuber melanosporum*. In addition, 3-methylbutanal could be detected in all the samples except for *Tuber magnatum pico*.

2,4-Dithiapentane has been specified for the tenth position as it is the most important aroma component in *Tuber magnatum pico*. In addition, this substance could be detected in the truffle oil.

DMS, in twelfth place, was chosen because it was detectable in all the samples measured. It is considered an important aroma component of many truffle varieties. In addition, DMS was identified by Talou et al. as the key substance for the detection of truffles by dogs.

The last substance is 3-methylthio-1-propanol as this substance could be a possible interfering component. 3-Methylthio-1-propanol is formed in, among other things, rotten organic material as a breakdown product of sulfur-containing amino acids. As sulfur components seem to play a major role in truffle aroma, this component was tested to obtain an indication of whether sulfur components in general would attract the dogs.

### 2.4. Experiment with Truffle Dogs

To check the marker substances selected in Table 2, an experiment was carried out with a total of 19 truffle dogs. One dog came from Germany, five came from the USA, and thirteen from Spain. The period since the dogs had been used as truffle dogs varied between four months and eight years. The breeds of the dogs also differed. The breeds represented were Golden Retriever, Beagle, Labrador, a cross between Beagle and Fox Terrier, Australian Kelpie, French Brittany, Border Collie, Lagotto Romagnolo, Spanish Water Dog, and Brittany Spaniel. In addition, the truffle types usually searched for by the truffle dog were queried. A total of 15 truffle farmers stated that the dogs usually search for *Tuber melanosporum*. Some animals also look for *Tuber aestivum*, *Tuber indicum*, *Tuber gibbosum*, *Tuber* uncinatum, or *Tuber borchii*.

**Figure 4:**
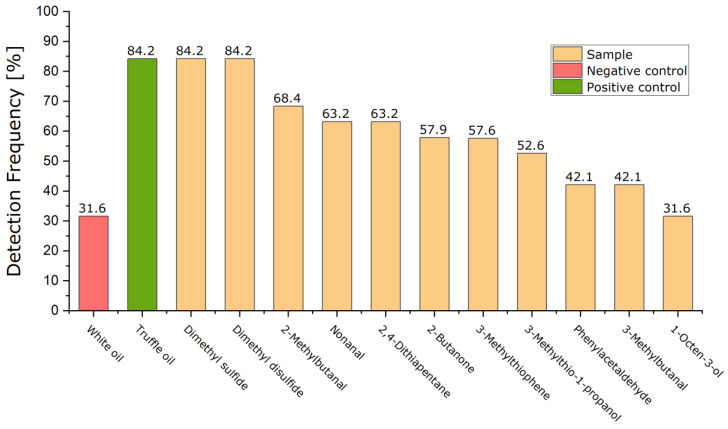
Detection frequencies of the tested substances.

As part of the experiment, the samples were applied individually and one after the other to a sponge, which acted as a carrier material, and buried in the ground. The dogs were then led past the sample and their reactions observed. If a dog identified a sample as a supposed truffle, it was noted. Table A2 and Table A3 in the Appendix A list all the feedback from the truffle farmers. Figure 4 graphically shows the results of the experiment based on the percentage detection frequencies.

**Figure 5:**
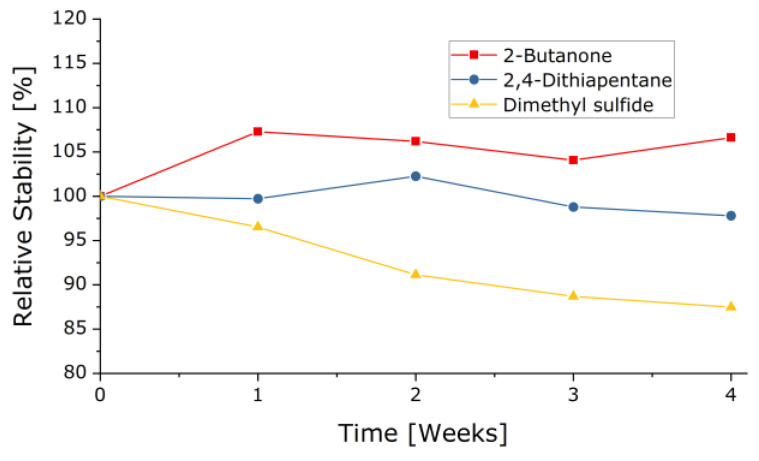
Stability study of selected sample compounds.

To ensure that the samples still had their original aroma intensity even after they had arrived at the truffle farmers, the stability of the samples of 2,4-dithiapentane, 2-butanone, and DMS was measured weekly over a period of four weeks. The samples were stored at room temperature. Figure 5 shows that the aroma strength of all three substances changed only slightly.

In principle, it can be seen from the presentation of the measurement results that each substance was recognized by at least 31.6% and a maximum of 84.2% of the dogs. Therefore, within the scope of this experiment, no substance can be completely ruled out. Contrary to expectations, the negative control was also identified as a truffle by 31.6% of the dogs.

**Figure A1:**
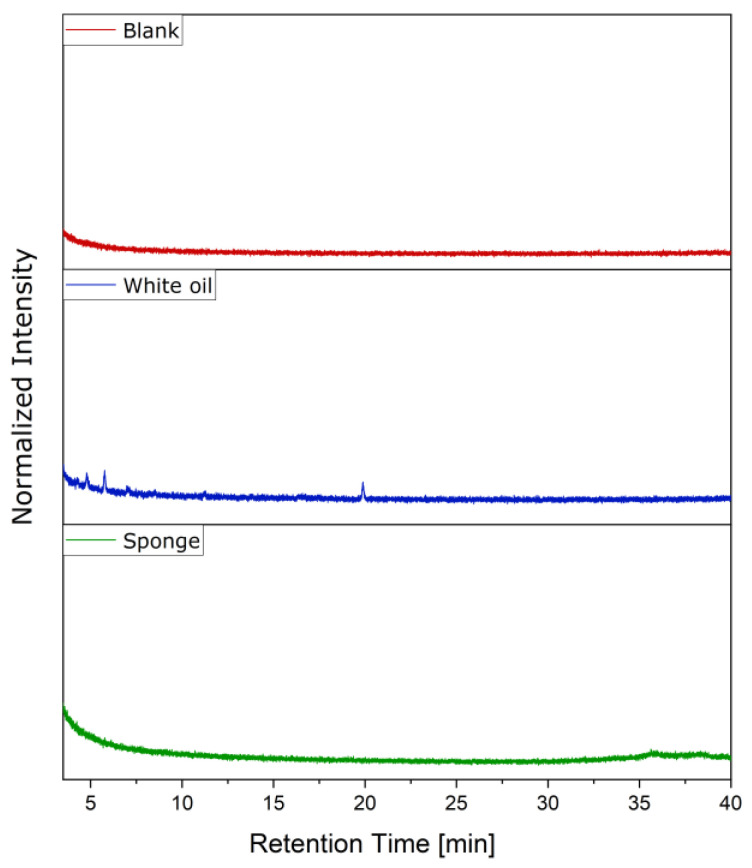
SHS/GC-MS total ion chromatograms of the blank measurement, white oil, and sponge.

As the occurrence of disturbing aroma components could previously be ruled out by the GC-MS measurements of the white oil and the sponges used (Appendix A, Figure A1), these results must have been influenced by other factors.

In addition to the positive control, the two substances, DMS and DMDS, have the highest percentage detection frequency at 84.2%. It can therefore be assumed that these two compounds are the best candidates as characteristic marker substances for truffles. No more precise assessment can be made for the other substances tested as all the substances have a detection frequency of between 31.6% and 68.4%. Even when comparing the detection frequencies with the origin, the breeds, and the period since the dogs were used as truffle dogs, no correlations could be found. There were no identical test protocols; so, it can also be said that all the dogs reacted individually to the samples.

When comparing the detection frequencies with the types of truffles usually searched for, no connections could be detected either as almost all the dogs usually search for *Tuber melanosporum*. To be able to investigate this further, in the future, it would be important that the dogs which usually look for *Tuber magnatum pico* also take part in the experiment as this truffle species was not represented among the participating dogs. Possibly, 2,4-dithiapentane would then also have a more meaningful detection frequency.

## 3. Discussion

Overall, the dog study results are less clear than could have been expected based on the literature and the SHS/GC-MS measurements. To be able to make further statements and to check the reproducibility of the data, the test would have to be carried out again, and any influencing factors that might have occurred would have to be examined separately.

It is possible, for example, that by burying the samples and leading the dogs past afterwards, some of them only followed the track of their owner or recognized their smell. This influencing factor could be a reason why the negative control was partially identified as a truffle. To avoid such measurement inaccuracies, the samples could be buried and only tested with the dogs one or more days later.

Another disruptive factor could also be the prevailing weather conditions as these can influence the smell perception of dogs. This could be another reason why the reported test results vary significantly. A follow-up experiment would therefore ideally have to be carried out with all dogs in the same area or under precisely described weather conditions. In practice, however, this is difficult to implement.

It is also not yet known how exactly dogs perceive the smell of something or how their training affects their ability to smell. It is unclear whether the dogs tend to follow the scent trail of one or more specific compounds or whether they perceive the concentration gradient of an aroma composition (fingerprint).

It is also conceivable that different dogs have trained themselves to track down truffles in different ways. For example, differences in breed (and thus also in the olfactory system), environmental influences such as background odors, temperature, wind speed, or socio-economic factors such as the training method ensure that different dogs perceive different substances when searching for truffles.

## 4. Materials and Methods

### 4.1. Chemicals and Materials

The VOC components phenylacetaldehyde, 1-octen-3-ol, 3-methylthiophene, 2-butanone, nonanal, dimethyl sulfide (DMS), 2-methylbutanal, 2,4-dithiapentane, 3-methylbutanal, dimethyl disulfide (DMDS), and 3-methylthio-1-propanol were all purchased from Sigma-Aldrich at >98% purity. The white oil (runny, paraffinum perliquidum, pharmaceutical quality, Chemdiscount), the synthetic sponges (Belle Vous “round synthetic sponge”), and the truffle oil were purchased from commercial vendors. The truffle oil was sold with an indication of the respective type of truffle (*Tuber melanosporum*). Samples of the truffle varieties *Tuber magnatum pico*, *Tuber melanosporum*, *Tuber aestivum*, and *Tuber indicum* were purchased online (Buon Gusto C.N.C e.K., Braunschweig, Germany, 2021 and Terra Ross Ltd., Berlin, Germany, 2021). Immediately upon receipt, the truffle samples were visually and sensorially checked for the authenticity of the variety and freshness.

### 4.2. Sample Preparation

To measure the truffle samples, the truffles were first cut into 0.5 mm-thin slices with a truffle slicer to facilitate the transition of the VOC into the gas phase. Each 0.5 g was weighed out and transferred to a hermetically sealed 10 mL headspace vial with a septum. To avoid degradation of the samples, they were examined on the day of delivery. To measure the sponge used as a carrier material, 0.1 g was weighed analogously due to its low density. A volume of 0.5 mL was taken from the truffle oil, the white oil, and the samples of 2,4-dithiapentane, 2-butanone, and dimethyl sulfide used for stability measurement. This was also placed in an airtight 10 mL headspace vial. The same samples were used for the stability measurements as for the experiment with the truffle dogs.

### 4.3. Gas Chromatography-Mass Spectrometry (GC-MS)

For the SHS/GC-MS measurements, the autosampler Gerstel MPS—Multi Purpose Sampler (Gerstel GmbH & Co. KG, Mülheim an der Ruhr, Germany) was used. All samples were transferred to the agitator at 40 °C for 15 min before injection. The samples were additionally shaken at 250 rpm with an on-off time of 10 s to 1 s. Even though the transition to the gas phase was stronger and the relative composition slightly different for even higher temperatures, 40 °C was chosen for these measurements. This was due to this being the lowest instrumentally possible temperature. Hence, this most resembles the conditions found in a natural environment and therefore what truffle dogs would smell. After the 15 min mentioned above, an equilibrium was achieved, and the concentrations of the individual components did not change anymore. The repeatability was checked with a triple detection of the same truffle (*Tuber melanosporum*). The standard deviation was less than 5%. To test the linearity, 5 samples of *Tuber melanosporum* weighing between 0.1 g and 2 g were measured. The linear regression coefficient amounted to 0.94.

The SHS measurements were performed using a syringe with a capacity of 2.5 mL. This was heated to 80 °C before sampling. The split injection was given over a period of 60 s with a split rate of 5:1. The injection volume was 1 mL at an injection temperature of 200 °C.

The gas chromatograph Agilent Technologies 7890B (Agilent Technologies Deutschland GmbH, Waldbronn, Germany) was equipped with a VF-624 ms column (60 m × 0.32 mm ID, 1.8 µm film thickness, Agilent). The column is specially designed for volatile and semi-volatile compounds. Helium 5.0 was used as the carrier gas (2.22 mL/min, constant flow). After the injection, the temperature of 40 °C was kept in the column oven for 15 min. This was followed by a temperature increase of 5 °C/min up to a temperature of 180 °C. Finally, the temperature was increased by 25 °C/min up to a temperature of 220 °C and held for 15 min, resulting in a total run time of 59.6 min.

The eluting substances were detected with an Agilent Technologies 5977A mass spectrometer (Agilent Technologies Deutschland GmbH, Waldbronn, Germany) using EI ionization at 70 eV. In scan mode, 30 *m*/*z* was set as the start mass and 250 *m*/*z* as the end mass. The analysis frequency was 6 scans/s at a speed of 1.562 u/s. The gain factor was set to 5 for the SHS measurements.

### 4.4. Data Analysis

The Chemstation software (Version C.01.05, Agilent Technologies Deutschland GmbH, Waldbronn, Germany) was used for the data evaluation. For identification, the fragmentation patterns of the individual peaks were compared with the NIST EI Library (Version NIST 11, National Institute of Standards and Technology, Gaithersburg, MD, USA). If the MS spectra matched >85%, the identification was considered positive. The retention order and the plausibility of the occurrence of the substance in the sample type were considered as soft decision criteria in the case of doubt. Retention indices for the stationary phase used could not be found in the literature.

### 4.5. Experiment with Truffle Dogs

**Figure 6:**
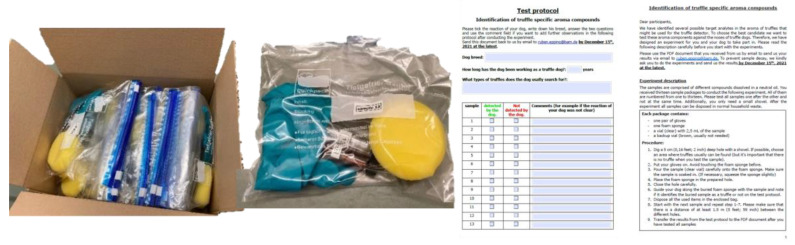
Sample package for the truffle dog experiment.

Each of the 19 participating truffle farmers from the United States, Spain, and Germany received a package containing 13 different, sealable sample bags, a set of test instructions, and a test protocol, as shown in Figure 6.

Each sample bag contained a pair of disposable gloves to avoid cross-contamination, 2.5 mL of the respective sample in a labeled 8 mL sample container made of clear glass, another sample as a fallback in an 8 mL amber glass sample container, and a yellow, round foam sponge with a diameter of 7.3 cm as carrier material. Except for the positive and negative controls, the sample solutions all had a concentration of 25 mg/L. The samples were prepared by dissolving the commercially purchased pure substances in white oil.

To carry out the experiment, a hole about five centimeters deep was to be dug for each sample in succession, starting with number 1, near the common truffle locations. Then, the gloves should be put on, and the contents of the sample from the sample container applied to the sponge. The sponge with the sample now had to be placed in the prepared hole. After plugging the hole, the dog should be walked along the buried specimen and its reaction observed. The dog’s reaction should then be noted on the test protocol and only after the tested sample had been disposed of should the subsequent one be started.

The response of the dogs was noted on the attached log. This contained a tabular listing of the sample numbers and an option to tick whether the dog recognized the sample or not. The query for the results was carried out in this (yes/no) form to ensure the comparability of the data. In addition, it was asked how long the dog had been active as a truffle dog, which types it usually looked for, and which breed it belonged to.

## 5. Conclusions

In conclusion, it can be said that the results obtained give a good indication of the marker substances of truffles. Presumably, the truffle aroma is mainly characterized and perceived by dogs by dimethyl sulfide and dimethyl disulfide and not by the other volatile organic compounds or sulfur-containing volatile organic compounds found in truffles. However, the factors mentioned in the discussion make it difficult to determine this with great certainty. As dogs are living being and not analytical instruments, they are influenced by a great variety of factors. Due to this, it seems unavoidable that one has to live with some degree of uncertainty regarding these data.

## Figures and Tables

**Table 1:** Specific aroma components of different truffle varieties according to literature.

*T. magnatum pico*	*T. melanosporum*	*T. aestivum*	*T. indicum*
2,4 Dithiapentane [3,20]	Anisole [3]	2-Butanone [3,5]	3-Methylanisole [3,5]
2,3-Butadione [20]	2-Methylbutyraldehyd [3,24]	2-Butanol [3,5]	
3-(Methylthio)propanol [20]	3-Methylbutanal [3,5]		
	2-Methylpropyl ethanoate [5]		

**Table 2:** Overview of the selected marker substances, their odor threshold values, literature reference for occurrence in truffles, and results from SHS/GC-MS measurements.

Nr.	Name	Structure	Odor Threshold [µg/L]	Literature/Description	SHS/GC-MS Measurements
1	Medical grade white oil	-	-	Odorless, negative control	-
2	Truffle oil	-	-	Positive control	-
3	Phenylacet-aldehyde	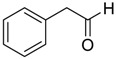	6100 [26]	[23]	Not detected
4	1-Octen-3-ol	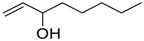	1–45 [26,30]	[3,5,23]	Truffle oil, possible ripeness marker
5	3-Methylthiophene	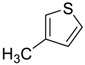	No literature references	[3,24]	Not detected
6	2-Butanone	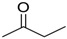		[3,5,28]	*T. melanosporum*, *T. aestivum*, *T. indicum*, truffle oil
7	Nonanal	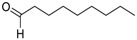		[3]	Truffle oil
8	Dimethyl disulfide	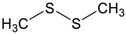	0.16–1.2 [31]	[3,5]	Not detected
9	2-Methylbutanal	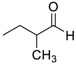	1.5 [26]	[3,5,22,23,28]	*T. melanosporum*
10	2,4-Dithiapentane	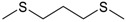	0.012 [26]	[3,5,22,23]	*T. magnatum pico*, truffle oil
11	3-Methylbutanal	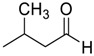	0.5–12 [26,30]	[3,5,23,24]	*T. melanosporum*, *T. aestivum*, *T. indicum*, truffle oil
12	Dimethyl sulfide	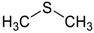	0.74 [26]	[3,5,22,23,24,28]	Detected in all samples
13	3-Methylthio-1-propanol	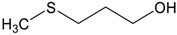	0.2 [26]	Possible interference component, originating from decomposing materials	

## Data Availability

Not applicable.

## Appendix A

**Table A1:** Identified aroma substances from SHS/GC-MS measurements as peak area percentages from entire chromatogram.

Name	Retention Time [min]	*T. magnatum pico*	*T. melanosporum*	*T. aestivum*	*T. indicum*	Truffle Oil
Acetaldehyde	3.51		6.00	0.12		
Methanethiol	3.77	1.18				33.2
Ethanol	4.86		3.39	0.07		12.3
Propanal	5.61		1.76			
Acetone	5.74		1.38	0.71	31.53	
Dimethyl sulfide	5.90	54.35	45.18	10.34	18.87	2.9
3-Methylpentane	7.69		0.07			
2-Methylpropanal	8.22		3.43	0.32		
Methyl formate	8.83		0.32		15.36	
1-Propanol	9.22		1.17			
2-Butanone	11.17		2.63	54.38	5.52	0.4
2-Butanol	12.18		0.21	33.13		
*p*-Dithian-2,5-diol	12.47					14.4
2-Methyl-1-propanol	15.51		1.76	0.03	15.94	
3-Methylbutanal	16.72		2.10	0.04	2.53	0.2
2-Methylbutanal	17.44		14.00	0.59		
sec-Butyl formate	18.37		13.88		10.25	
sec-Butyl acetate	24.03			0.15		
2,2-Dimethylcyclobutanone	24.36			0.12		
2-Methyl-1-butanol	24.38		2.49			
2-Methylbutyl formate	26.55		0.08			
Hexanal	27.15		0.04			
2,4-Dithiapentan	31.37	44.46				30.9
1,3-Octadiene	35.36					4.6
Nonanal	39.43					1.1

**Table A2:** Results protocol of the experiment with dogs.

Sample	Dog Nr.																		
	1	2	3	4	5	6	7	8	9	10	11	12	13	14	15	16	17	18	19
White oil	no	no	yes	yes	no	yes	no	no	yes	no	no	no	yes	no	no	no	no	no	yes
Truffle oil	no	no	yes	no	yes	yes	yes	yes	yes	yes	yes	yes	yes	yes	yes	yes	yes	yes	yes
Phenylacetaldehyde	no	no	yes	yes	no	yes	yes	no	yes	no	yes	no	yes	no	no	no	no	yes	no
1-Octen-3-ol	yes	yes	no	no	no	yes	no	no	yes	no	no	yes	yes	no	no	no	no	no	no
3-Methylthiophene	no	yes	yes	no	no	yes	yes	yes	no	no	no	yes	no	yes	yes	no	yes	yes	yes
2-Butanone	yes	yes	yes	no	no	yes	yes	yes	no	no	yes	yes	no	no	yes	no	yes	yes	yes
Nonanal	yes	yes	yes	no	no	yes	no	yes	yes	no	no	yes	no	no	yes	yes	yes	yes	no
Dimethyl disulfide	yes	yes	yes	yes	no	yes	yes	yes	no	yes	yes	no	yes	yes	yes	yes	yes	yes	yes
2-Methylbutanal	yes	yes	yes	no	yes	no	yes	yes	yes	yes	yes	yes	yes	no	no	no	no	yes	yes
2,4-Dithiapentane	no	no	yes	no	no	yes	yes	yes	no	no	yes	yes	yes	yes	yes	no	yes	yes	yes
3-Methylbutanal	yes	no	no	no	no	yes	no	no	yes	no	yes	yes	yes	yes	no	no	no	no	yes
Dimethyl sulfide	yes	yes	yes	no	yes	yes	yes	yes	yes	no	yes	yes	yes	yes	no	yes	yes	yes	yes
3-Methylthio-1-propanol	yes	yes	yes	no	no	yes	yes	no	no	no	no	yes	no	no	no	no	yes	yes	yes

**Table A3:** Additional information on the truffle dogs.

Dog Nr.	Breed	Origin	Used as a Truffle Dog for	Truffle Variety
**1**	Beagle	Spain	5 Years	*T. melanosporum, T. aestivum*
**2**	Australian Kelpie	Spain	4 Years	*T. melanosporum, T. aestivum*
**3**	Labrador	Germany	8 Months	*T. melanosporum, T. uncinatum*
**4**	French Britney	USA	1 Year	*T. gibbosum*
**5**	beagle x foxterrier	Spain	4 Years	*T. melanosporum, T. aestivum*
**6**	Ragotto Romagnolo	Spain	4.5 Years	*T. melanosporum, T. aestivum*
**7**	Spanischer Wasserhund	Spain	2 Years	*T. melanosporum*
**8**	Spanischer Wasserhund	Spain	2 Years	*T. melanosporum*
**9**	Border collie	Spain	2.5 Years	*T. melanosporum, T. aestivum*
**10**	Lagotto Romanolo	Spain	2 Years	*T. melanosporum, T. aestivum, T. indicum*
**11**	Lagotto Romagnolo	Spain	2 Years	*T. melanosporum*
**12**	No information	Spain	No information	No information
**13**	Golden Retriever	Spain	4 Years	*T. melanosporum*
**14**	No information	USA	No information	No information
**15**	Lagotto Romagnolo	USA	8 Years	*T. melanosporum, T. borchii, T. aestivum, T. uncinatum*
**16**	Lagotto Romagnolo	USA	4 Months	*T. melanosporum, T. borchii, T. aestivum, T. uncinatum*
**17**	Brittany Spaniel	USA	5 Years	*T. melanosporum, T. borchii, T. aestivum, T. uncinatum*
**18**	No information	Spain	No information	No information
**19**	No information	Spain	No information	No information
